# Identification of clinical implications and potential prognostic models of chromatin regulator mutations in multiple myeloma

**DOI:** 10.1186/s13148-022-01314-7

**Published:** 2022-07-23

**Authors:** Lina Zhang, Run Zhang, Jing Wang, Ying Chen, Chun Qiao, Qinglin Shi, Yuanyuan Jin, Xuxing Shen, Jianyong Li, Lijuan Chen

**Affiliations:** Department of Hematology, The First Affiliated Hospital of Nanjing Medical University, Jiangsu Province Hospital, Collaborative Innovation Center for Cancer Personalized Medicine, Nanjing, 210029 China

**Keywords:** Next-generation sequencing, Chromatin regulator, Epigenetic, Multiple myeloma, Nomogram

## Abstract

**Background:**

With the rapid development of next-generation sequencing (NGS) technologies, researchers are making efforts to reveal the genomic landscape of multiple myeloma (MM). However, the clinical significance of many mutations remains poorly defined due to the genetic heterogeneity of MM. To systematically explore the clinical implications of gene mutations and build practical prognostic models, we performed DNA sequencing in newly diagnosed MM patients.

**Methods:**

MM cells were purified from bone marrow aspirates using CD138 microbeads and subjected to sequencing with a 387-gene Panel. Nomogram was developed using Cox’s proportional hazards model, and candidate variables were screened by stepwise regression. Internal validation was carried out by the bootstrap method.

**Results:**

Between July 2016 and December 2020, a total of 147 patients were included in our study. We found patients with a higher mutational load had a significantly shorter progress-free survival (PFS) (19.0 vs. 32.0 months, *P* = 0.0098) and overall survival (OS) (3-year OS rates were 66.1% and 80.0%, *P* = 0.0290). Mutations in chromatin regulators (CRs) including *KMT2C* (14.3%), *KMT2D* (14.3%), *EP300* (11.6%) and ARID gene family (31.3%) were highly frequent in newly diagnosed MM patients. Interestingly, proteins encoded by these genes could form a complex called KMT2C/D COMPASS (K_CD_COMs). Patients with mutations of ARID gene family had a significantly shorter PFS (15.5 vs. 34.0 months, *P* = 0.0003) and OS (3-year OS rates were 64.9% and 81.0%, *P* = 0.0351) than patients without ARID gene mutations. Incorporating ARID gene mutations into the current staging system could successfully improve their prognostic performance. The PFS and OS nomogram models (including 1q21 copies, ARID gene mutations, extramedullary disease, mutational load and *TP53* mutations) showed good predicting performance in both training and validation sets.

**Conclusion:**

Our findings emphasized the importance of CRs mutations in newly diagnosed MM patients and indicated the mutations affecting K_CD_COMs might promote the development of MM. High mutational load and harboring mutations in the ARID gene family were novel predictors of adverse prognosis in MM. Prognostic models based on gene mutations were commendably prognostic evaluation methods that could provide a reference for clinical practices.

**Supplementary Information:**

The online version contains supplementary material available at 10.1186/s13148-022-01314-7.

## Introduction

Multiple myeloma (MM) is a common hematological malignancy characterized by neoplastic proliferation of monoclonal plasma cells [1]. Despite the emergence of numerous new drugs, it is still an incurable disease. Therefore, the biological characteristics of MM require further exploration to find new predictive markers and therapeutic approaches. The advent of next-generation sequencing (NGS) technologies has provided a powerful tool for studies on cancer genetics in the past 15 years. MM showed a moderate level of tumor mutational load (1.6 mutations/Mb) across various cancer types, which suggested an important role of gene mutations in the occurrence and development of MM [2].

Several studies [3–5] have explored the genetic mutation profile of MM using NGS technologies since 2014, and the authors were consistent with the conclusion that the top recurrent mutated genes were *KRAS* (mutation rates, 20–23%) and *NRAS* (19–20%) in MM. Other common mutated genes included *DIS3*, *FAM46C*, *BRAF*, *TP53* and *TRAF3*. These genes with a higher mutation rate were concentrated in five pathways, including the MAPK pathway (*KRAS*, *NRAS* and *BRAF*), plasma cell differentiation pathway (*IRF4* and *PRDM1*), the NF-κB pathway (*TRAF3*, *CYLD* and *LTB*), cell-cycle control pathway (*RB1* and *CCND1*) and DNA repair pathway (*ATM*, *ATR*, *TP53*) [5, 6]. Previous studies confirmed that gene mutations also had considerable clinical and prognostic significance in MM. Mutations of *IRF4* and *EGR1* were found to be correlated with a favorable prognosis, while mutations of *TP53*, *ATM* and *ATR* were associated with a poor prognosis [4–6]. However, the genetics of MM are complex and the clinical significance of many mutations remains unknown.

In 2020, Ordoñez et al. [7] compared malignant plasma cells versus normal B cells using a multi-epigenomics approach and found extensive activation of regulatory elements in tumor cells, which led to the overexpression of members involved in the NOTCH, NF-κB, mTOR and p53 signaling pathways and promoted the proliferation of plasma cells. These data demonstrated epigenetic changes played an important role in MM pathogenesis. Epigenetics refers to the regulation of gene expression without changes in DNA sequence, and chromatin regulators (CRs) are important research objects in epigenetics [8]. Based on the different functions, CRs can be grouped into three classes: (1) DNA methylators, including DNMT1, DNMT3A and DNMT3B; (2) histone-modifying enzymes, including KMT2C, KMT2D, p300 and HAT1; (3) chromatin remodelers, including ARID1A, ARID1B, ARID2, CHD5 and ACF1 [9]. Although mutations of CRs are common across multiple cancers, we still have limited knowledge of their roles in MM.

To further investigate the clinical significance of gene mutations in MM, we performed DNA sequencing in 147 patients with newly diagnosed multiple myeloma (NDMM) by NGS technologies and found mutations of CRs have important prognostic significance. We also developed nomogram models based on mutations of CRs to predict the risk of relapse or mortality in MM patients, which can be used conveniently in clinical practice.

## Materials and methods

### Patients and data collection

The study was approved by the Ethics Committee of The First Affiliated Hospital of Nanjing Medical University. The use of tumor specimens was approved by the Ethics Committee, and informed consent was obtained from all patients. The diagnoses of patients were made based on the International Myeloma Working Group 2014 criteria [10]. Clinical staging was based on the Durie–Salmon (DS) staging system [11], International Staging System (ISS) and Revised International Staging System (R-ISS) [12].

Laboratory examination data such as hemoglobin (Hb), serum creatinine, bone marrow plasma cells proportion, lactate dehydrogenase (LDH) and β_2_ microglobulin (β_2_-MG) were collected in our study. The presence of extramedullary disease (EMD) was detected by PETCT, whole-body CT or whole-body MRI. High-risk cytogenetic abnormalities were defined as at least one of the following: del(17p), t(4; 14), or t(14; 16) using cytoplasmic immunoglobulin fluorescence in situ hybridization (cIg-FISH). The numerical aberrations of the chromosomal regions 1q21 were also detected by cIg-FISH. Progress-free survival (PFS) was calculated from the time of diagnosis to disease progression (PD) or death due to any reason. Overall survival (OS) was estimated as the time from diagnosis to death.

### Next-generation sequencing

Bone marrow samples were collected before treatment, and tumor cells were purified by positive selection with anti-CD138 magnetic microbeads (Miltenyi Biotec, Germany). Genomic DNA was extracted from tumor cells using the QIAamp® Blood DNA Mini Kit (QIAGEN, Germany). The quantity of DNA was measured using a Qubit 2.0 fluorometer (Life Technologies, USA). Genotyping was performed using a sequencing panel of 387 genes (see Additional file 2 for details) by NGS technologies, and the average sequencing depth was 1000×. Genomic DNA (200 ng) was sheared with Enzyme Plus Library Prep Kit (iGenetech, China) and sequencing libraries were constructed using probes and TargetSeq One Kit (iGenetech, China) according to the instructions. Sequencing runs were performed on NovaSeq 6000 sequencing platform (Illumina, USA) using a NovaSeq 6000 S4 Reagent Kit v1.5 (300 cycles) (Illumina, USA). The raw sequencing data were available in the SRA database (https://www.ncbi.nlm.nih.gov/sra) under the accession number PRJNA826654.

### Analysis of mutations

FASTQ files were pre-processed and assessed for quality with fastp. Sequencing reads were aligned to the human reference genome hg19 using BWA v. 0.7.17 software. SAMtools v. 1.11 software was used to convert Sequence Alignment/Map (SAM) files to Binary Alignment Map (BAM) [13], and gatk v.4.1.3.0 was applied to perform indel realignment and base quality recalibration. BAM files were sorted with SortSam, duplicate reads were removed with gatk MarkDuplicates, mapped reads were extracted with SAMtools, and base quality score was recalibrated with gatk BaseRecalibrator and ApplyBQSR [14]. Subsequently, we annotated the variants using Annovar and SnpEff [15]. Single nucleotide polymorphisms (SNPs) were identified using information from ExAC_ALL, ExAC_EAS and 1000g2015aug_all databases, and they were excluded from further analysis.

### Derivation and validation of predictive nomogram models

The predictive nomogram was developed using Cox’s proportional hazards model. Candidate variables were screened by stepwise regression. For the model fitting, we used the *R* package “survival”. Model diagnostics were then performed by package “survminer”, including Deviance residues to assess the effect of outlier cases and Schoenfeld residuals to test the proportional risk assumption. The area under the receiver operator curve (ROC) (AUC/C-statistic) and Brier score were calculated to assess the performance of the model. Finally, the model was retested for internal validation using the bootstrap method, with 100 replications.

### Statistical methods

Statistical analysis and plotting were performed by *R* software v. 4.1.1 and GraphPad Prism v. 5.0. Numerical variables were analyzed by *t*-test or ANOVA, and non-normal distribution variables were analyzed by Mann–Whitney *U* test or Kruskal–Wallis test. Categorical variables were analyzed using Chi-square test. K–M method and Log-rank test were used to plot and analyze the survival curves. Cox proportional hazards model was used for multifactorial analysis. For all analyses, a *P* value less than 0.05 was considered significant.

## Results

### Clinical characteristics of our study population

From July 2016 to December 2020, a total of 147 NDMM patients were included in the study and the clinical characteristics of patients are summarized in Table 1. The median age was 63.0 years (range, 38.0–84.0 years), and 60.5% of patients were male. The majority of patients (50.3%) were treated with proteasome inhibitors (PIs)-based regimen as first-line chemotherapy, such as PCD (bortezomib/cyclophosphamide/ dexamethasone), PAD (bortezomib/doxorubicin/dexamethasone) and VD (bortezomib/dexamethasone). 38.1% of patients received combination therapy with PIs and immunomodulatory drugs (IMiDs), 9.5% of patients received IMiDs-based regimen, and the remaining three patients were treated symptomatically.

**Table 1:** Clinical characteristics of 147 patients

Clinical characteristics	Proportion (%)
*Gender*	
Male	60.5 (89/147)
Female	39.5 (58/147)
*Subtype*	
IgG	48.3 (71/147)
IgA	24.5 (36/147)
IgD	6.1 (9/147)
IgE	0.7 (1/147)
κ light chain	11.6 (17/147)
λ light chain	8.8 (13/147)
*DS stage*	
I	4.1 (6/147)
II	13.6 (20/147)
III	82.3 (121/147)
*ISS stage*	
I	15.6 (23/147)
II	32.0 (47/147)
III	52.4 (77/147)
R-ISS stage*	
I	13.0 (18/138)
II	64.5 (89/138)
III	22.6 (31/138)
1q21*	
Gain	25.7 (35/136)
Amplification	24.3 (33/136)
High-risk karyotype*	
del(17p)	12.5 (17/136)
t(4; 14)	17.6 (24/136)
t(14; 16)	0.7 (1/136)
Anemia^†^	64.6 (95/147)
Renal insufficiency^‡^	31.3 (46/147)
Extramedullary disease	20.4 (30/147)
*Induction therapy regimen*	
PIs-based	50.3 (74/147)
IMiDs-based	9.5 (14/147)
PIs + IMiDs	38.1 (56/147)
Completed ASCT	25.9 (38/147)

*ISS* International Staging System, *PIs* proteasome inhibitors, *IMiDs* immunomodulatory drugs, *ASCT* autologous hematopoietic stem cell transplantation

*Some patients did not undergo the FISH examinations, so the proportions are calculated by the numbers of patients with FISH results

^†^Anemia refers to hemoglobin < 100 g/L

^‡^Renal insufficiency refers to creatinine > 177 µmol/L or creatinine clearance < 40 ml/min

Follow-up time ranged from 2.0 to 67.0 months, with a median time of 26.0 months. During follow-up, 49.7% of patients developed PD and 22.4% of patients died. The 1-year, 2-year and 3-year PFS rates were 77.3%, 56.9% and 37.4%, respectively (the median PFS was 28.0 months). The 1-year, 2-year and 3-year OS rates were 90.7%, 82.3% and 75.4%, respectively (the median OS was not reached).

### Patients with heavier mutational load had a poor prognosis

**Fig. 1:**
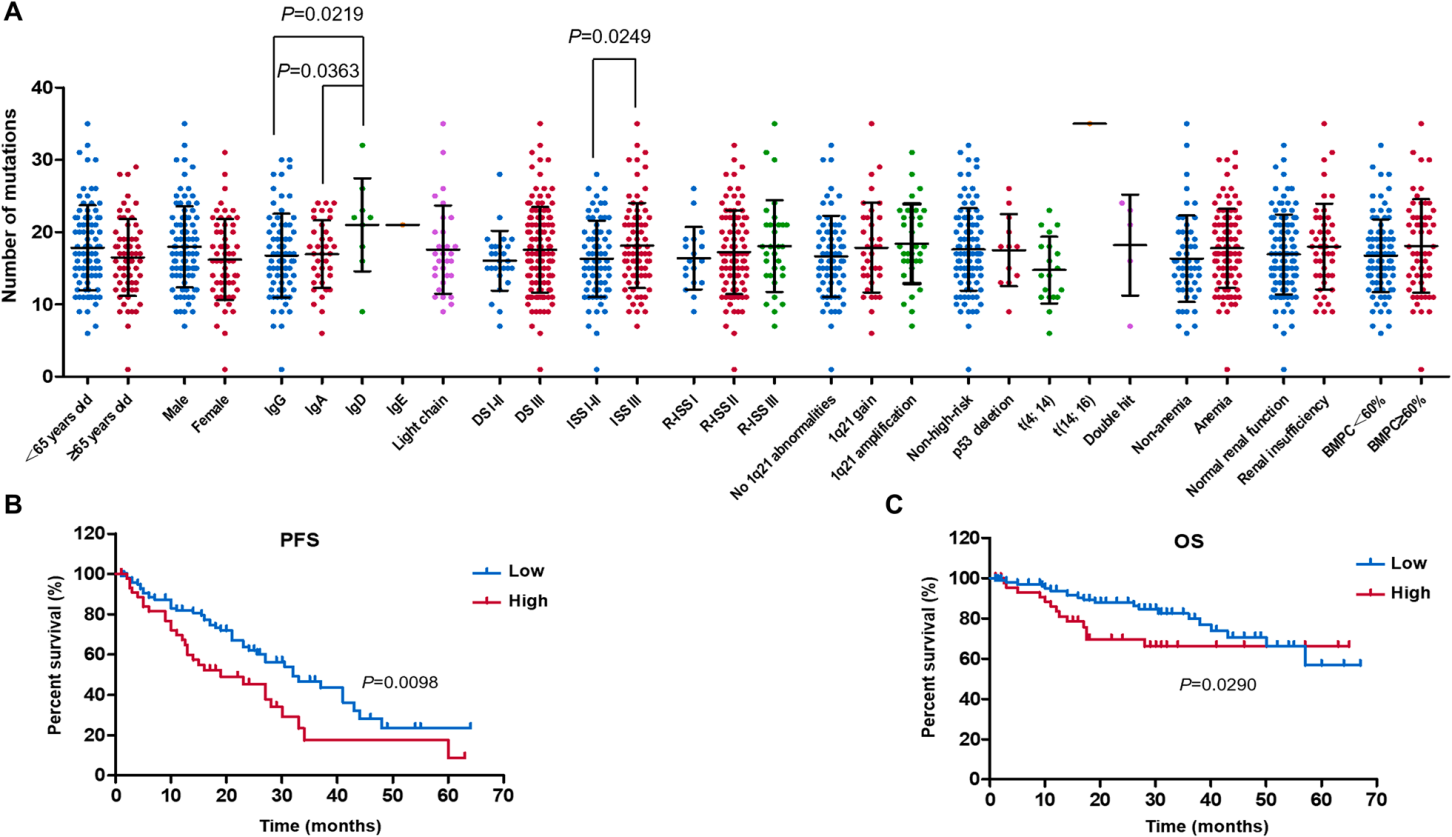
Clinical values of mutational load. **a** The mutational load of patients with IgD MM is significantly greater compared to IgG and IgA MM. Patients belong to ISS stage III have heavier mutational load than patients belong to ISS stage I and stage II. **b, c** Patients with high mutational load have a significantly shorter PFS and OS than patients with low mutational load

A total of 343 mutated genes were detected, and missense mutation was the most common mutation type. The median total number of mutations in each patient was 17.0 (range 1.0–35.0). In order to fully understand the clinical value of mutational load, we performed an integrated analysis of clinical features and the number of mutations (Fig. 1a). The number of mutations in patients with IgD MM was significantly greater compared to IgG (*P* = 0.0219) and IgA (*P* = 0.0363) MM. Patients with ISS stage I and stage II were combined because of the low number of patients in ISS stage I group. The mutational load of patients with ISS stage III was significantly heavier than those of patients with ISS stage I and stage II (*P* = 0.0249).

With 20 as the threshold, patients were divided into a high mutational load group (number of mutations ≥ 20, n = 47) and a low mutational load group (number of mutations < 20, n = 100). Patients in the low mutational load group had a significantly longer PFS than patients in the high mutational load group (median PFS, 32.0 vs. 19.0 months, *P* = 0.0098, Fig. 1b). In a similar manner, OS of the low mutational load group was significantly longer than that of the high mutational load group (median OS was not reached, 3-year OS was 80.0% and 66.1%, *P* = 0.0290, Fig. 1c). These results indicated that a high mutational load was associated with poor prognosis in MM patients.

### Profile of gene mutations

**Fig. 2:**
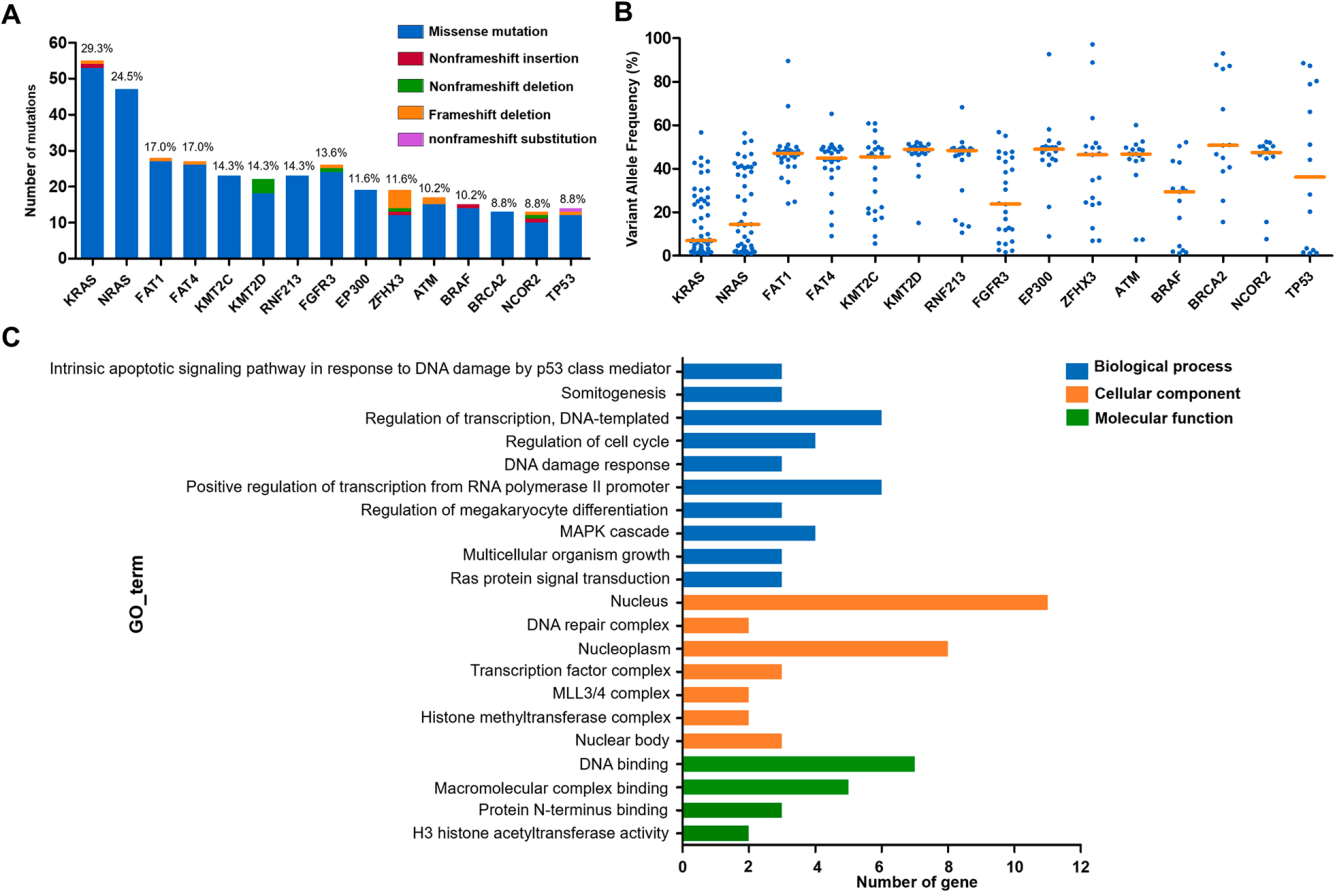
The most common gene mutations in patients with newly diagnosed multiple myeloma. **a** The mutation rates and types of the top 15 genes in our study. **b** The variant allele frequencies of the top 15 genes. **c** GO enrichment of the top 15 genes

In this study, the top 15 genes with the highest mutation frequencies were *KRAS* (29.3%), *NRAS* (24.5%), *FAT1* (17.0%), *FAT4* (17.0%), *KMT2C* (14.3%), *KMT2D* (14.3%), *RNF213* (14.3%), *FGFR3* (13.6%), *EP300* (11.6%), *ZFHX3* (11.6%), *ATM* (10.2%), *BRAF* (10.2%), *BRCA2* (8.8%), *NCOR2* (8.8%) and *TP53* (8.8%) (Fig. 2a). The variant allele frequencies (VAFs) are presented in Fig. 2b, and the median VAFs for each gene were *KRAS* (7.0%, range 1.0–56.7%), *NRAS* (14.4%, range 1.0–56.4%), *FAT1* (47.1%, range 24.0–89.6%), *FAT4* (44.8%, range 9.0–65.3%), *KMT2C* (45.5%, range 5.6–60.9%), *KMT2D* (48.9%, range 15.1–52.2%), *RNF213* (48.4%, range 10.6–68.3%), *FGFR3* (23.8%, range 1.7–56.9%), *EP300* (49.0%, range 8.9–92.7%), *ZFHX3* (46.4%, range 6.9–97.2%), *ATM* (46.8%, range 7.4–60.1%), *BRAF* (29.4%, 1.2–52.2%), *BRCA2* (50.8%, range 15.5–93.0%), *NCOR2* (47.4%, range 7.6–52.4%) and *TP53* (36.2%, range 1.2–88.5%).

To better understand the biological functions of the top 15 genes, we performed gene ontology (GO) enrichment analysis using DAVID (https://david.ncifcrf.gov/) (Fig. 2c). In biological process (BP) category, the mutated genes were significantly enriched in the terms “regulation of transcription, DNA-templated” and “positive regulation of transcription from RNA polymerase II promoter”. Correspondingly, these genes were markedly enriched in “nucleus” of cellular component (CC) category and “DNA binding” of molecular function (MF) category. The enrichment results indicated that genes involved in regulation of gene transcription (*KMT2D*, *ZFHX3*, *KMT2C*, *EP300*, *BRCA2* and *TP53*) might play an important role in MM pathogenesis. Interestingly, among the enriched genes, *KMT2D*, *KMT2C* and *EP300* belong to CRs and are implicated in the formation of KMT2C/D COMPASS complex (K_CD_COMs) [16].

### Correlation between chromosome abnormalities and mutated genes

The most common cytogenetic abnormalities in MM include copy number gains of 1q21, del(17p) as well as IgH translocations t(4; 14), t(11; 14), t(14; 16) and t(14; 20). However, only t(4; 14) was included in the further analysis for there were very few patients with t(11;14), t(14;16) and t(14;20).

**Fig. 3:**
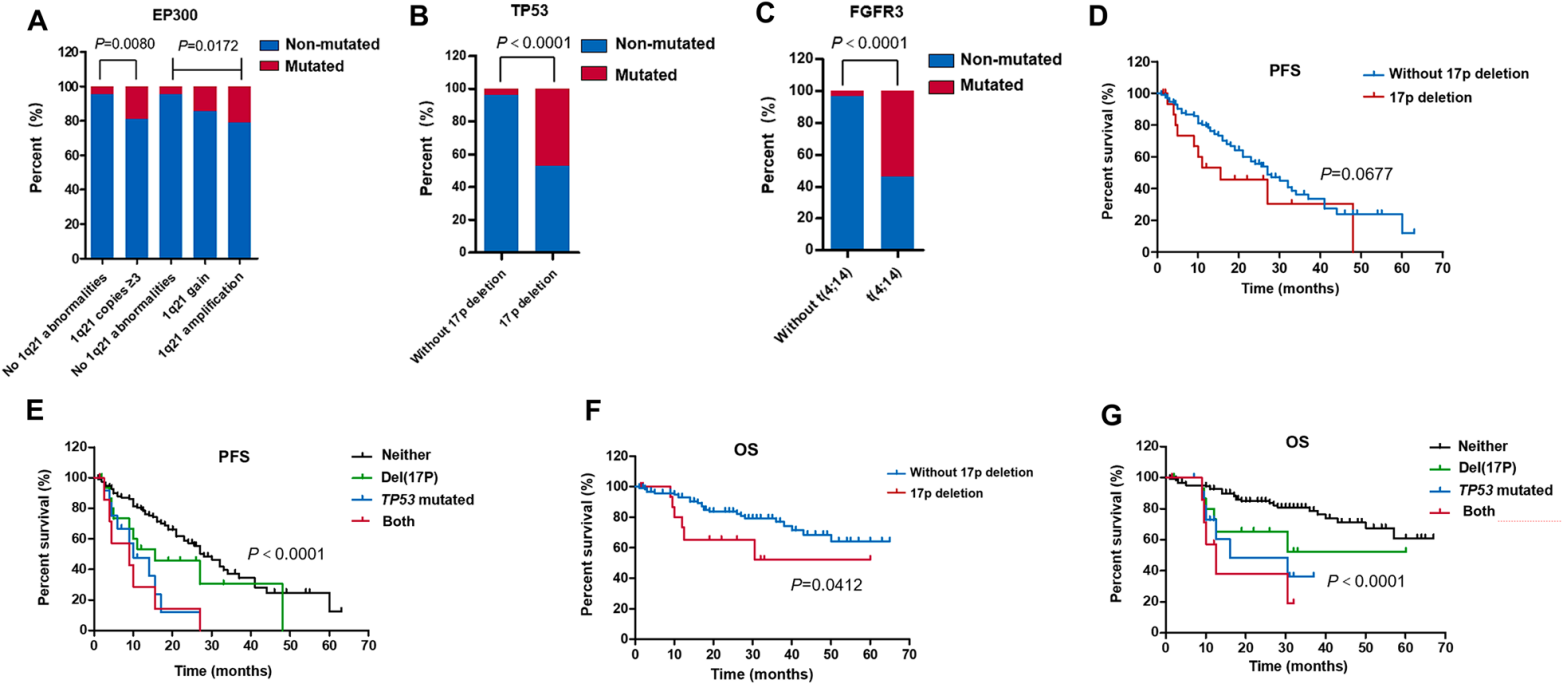
The correlation between chromosome abnormalities and gene mutations. **a** The increased mutation rates of *EP300* are associated with copy number gains of 1q21. **b** Patients with del(17p) have more mutations of *TP53*. **c** Mutation rates of FGFR3 increase in patients with t(4; 14). **d** Patients with del(17p) (n = 17) present shorter PFS compared to patients without del(17p) (n = 119), but this does not reach statistical significance. **e** Patients with both del(17p) and *TP53* mutations present the worst PFS compared to patients with only one of them. Patients with none of del(17p) or *TP53* mutations have the longest PFS. **f** Patients with del(17p) (n = 17) have shorter OS compared to patients without del(17p) (n = 119). **g** Patients with both del(17p) and *TP53* mutations present the worst OS compared to patients with only one of them. Patients with none of del(17p) or *TP53* mutations have the longest OS

The increased mutation rate of *EP300* (19.1% vs. 4.4%, *P* = 0.0080) was associated with copy number gains of 1q21. Three copies of 1q21 were defined as 1q21 gain, and four or more copies were defined as 1q21 amplification. With the increase of 1q21 copies, the mutation rates of *EP300* also gradually increased (the mutation rates were 4.4%, 14.3% and 21.2%, respectively; *P* = 0.0172, Fig. 3a). Moreover, there was a clear correlation between gene mutations and chromosome abnormalities on which the gene was located. Patients with del(17p) had noticeably more mutations of *TP53* (47.1% vs. 4.2%, *P* < 0.0001) (Fig. 3b) and mutation rates of *FGFR3* obviously increased in patients with t(4; 14) (54.2% vs. 3.6%, *P* < 0.0001) (Fig. 3c).

The prognostic value of chromosome abnormalities and gene mutations was evaluated using Kaplan–Meier survival analysis. In patients with del(17p), there was a tendency toward a reduction in PFS, but this did not reach statistical significance (median PFS, 15.5 vs. 27.0 months, *P* = 0.0677) (Fig. 3d). We re-grouped patients into four groups according to del(17p) and *TP53* mutations: neither group contained patients without del(17p) and *TP53* mutations (n = 114), del(17p) group contained patients with del(17p) positive (n = 17), *TP53* mutated group contained patients harboring *TP53* mutations (n = 13), and both group contained patients harboring del(17p) and *TP53* mutations concomitantly (n = 8). The median PFS for these four groups were 28.0, 15.5, 10.0 and 9.0 months. A significant difference in PFS among the four groups was observed (*P* < 0.0001) (Fig. 3e), which suggested that patients could be better stratified by the combined use of del(17p) and *TP53* mutations. Analysis for OS was performed next in the same manner. Patients with del(17p) had shorter OS compared to patients without del(17p) (median OS was not reached, 3-year OS was 52.1% and 76.9%, *P* = 0.0412) (Fig. 3f). The 3-year OS for neither group, del(17p) group, *TP53* mutated group and both group were 77.1%, 52.1%, 36.4% and 19.0%, respectively (*P* < 0.0001) (Fig. 3g). Similar to the PFS, the combined use of del(17p) and *TP53* mutations allowed a better prediction of OS. Moreover, our results showed the effect of *TP53* mutations on patients’ prognosis was more pronounced than del(17p). However, further studies were needed to draw the final conclusion. Combined analysis of 1q21 gain and *EP300* mutations, t(4; 14) and *FGFR3* mutations did not show better discriminating abilities for predicting the outcomes of patients.

### Mutations in ARID gene family were potential indicators of a poor prognosis

**Fig. 4:**
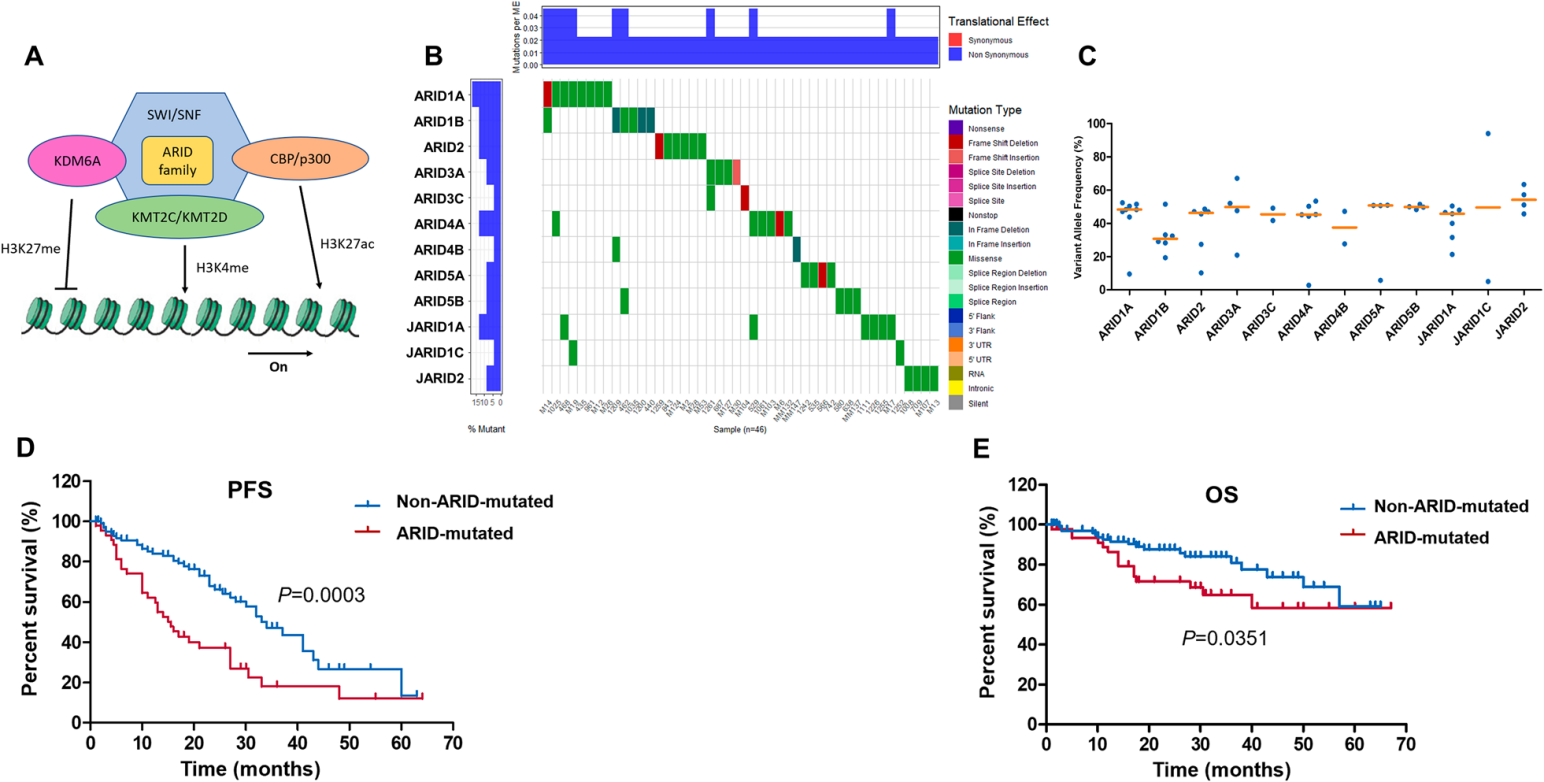
Mutations of ARID gene family. **a** Schematic illustration of K_CD_COMs structure: KMT2C, KMT2D, p300 (encoded by EP300) and ARID family are components of K_CD_COMs. KMT2C/KMT2D are involved in the methylation of H3K4, p300 regulates H3K27 acetylation, and ARID family participates in chromatin remodeling. The activated K_CD_COMs can promote transcription. **b** Waterfall plot of mutations in ARID family. **c** The variant allele frequency of mutations in ARID family. **d** The PFS of patients with ARID gene family mutations is significantly shorter than patients without ARID gene mutations. **e** The OS of patients with ARID gene family mutations is significantly shorter than patients without ARID gene mutations

K_CD_COMs complex might play an important role in the development of MM as previously shown. Unfortunately, survival analysis found no difference in PFS or OS between patients with and without mutations of *KMT2C*, *KMT2D* or *EP300*. Therefore, we focused on another component in this complex, called ARID family (Fig. 4a). In our study population, 46 patients (31.3%) had one or more mutations in ARID gene family and missense mutation was the most common mutation type (Fig. 4b). The median VAFs for each gene were *ARID1A* (48.3%, range 9.5–52.3%), *ARID1B* (30.7%, range 19.3–51.5%), *ARID2* (46.3%, range 10.2–48.6%), *ARID3A* (49.8%, range 20.9–67.1%), *ARID3C* (45.5%, range 41.7–49.2%), *ARID4A* (45.2%, range 2.7–53.4%), *ARID4B* (37.4%, range 27.7–47.2%), *ARID5A* (50.7%, range 5.8–50.9%), *ARID5B* (49.9%, range 48.3–51.4%), *JARID1A* (45.7%, range 21.3–50.4%), *JARID1C* (49.6%, range 5.0–94.1%) and *JARID2* (54.1%, range 45.6–63.4%) (Fig. 4c).

To better understand the effects of ARID gene family on the prognosis of MM, we compared PFS and OS between patients with and without mutation of ARID gene family. We divided patients into two groups: the ARID mutated group (n = 46) referred to patients who carried mutations of ARID family and the non-ARID-mutated group (n = 101) referred to patients without mutations of ARID family. PFS was significantly shorter in the ARID mutated group compared to the non-ARID-mutated group (median PFS, 15.5 vs. 34.0 months, *P* = 0.0003) (Fig. 4d). Similarly, the ARID mutated group had obviously shorter OS than the non-ARID-mutated group (median OS was not reached, 3-year OS was 64.9% and 81.0%, *P* = 0.0351) (Fig. 4e).

To further evaluate the prognostic significance of ARID family mutations, we combined them with ISS and R-ISS staging system. Patients who belonged to ISS/R-ISS stage I and did not have mutations of ARID family were still divided into stage I; patients who belonged to ISS/ R-ISS stage III and have mutations of ARID family were divided into stage III; all other situations were divided into stage II (Table 2).

**Table 2:** ARID gene mutation staging system

Stage	ISS + ARID	R-ISS + ARID
I	ISS stage I without ARID mutations*	R-ISS stage I without ARID mutations
II	Not stage I or stage III	Not stage I or stage III
III	ISS stage III with ARID mutations	R-ISS stage III with ARID mutations

*ISS* international staging system, *R-ISS* revised international staging system

*ARID mutations refer to gene mutations in ARID gene family

**Fig. 5:**
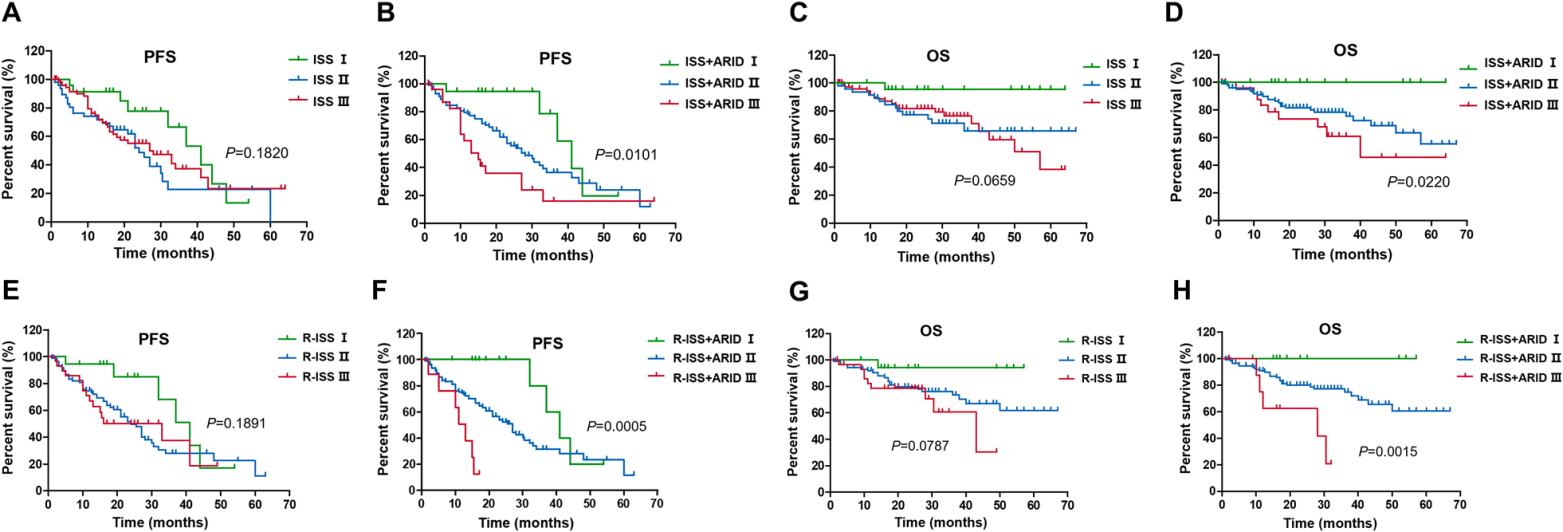
ISS + ARID and R-ISS + ARID staging system. **a** The PFS of patients cannot be precisely distinguished using ISS staging system. **b** A significant reduction of PFS is observed as the ISS + ARID stage progressed. **c** OS cannot be clearly distinguished according to ISS staging system. **d** There is a significant difference of OS by using ISS + ARID staging system. **e** There is no significant difference in PFS using R-ISS staging system. **f** A significant reduction of PFS is observed as the R-ISS + ARID stage progressed. **g** OS cannot be clearly distinguished according to R-ISS staging system. **h** There is a significant difference of OS by using ISS + ARID staging

According to the ISS, 23 patients were stage I, 47 patients were stage II, and 77 patients were stage III. The PFS could not be precisely distinguished (median PFS were 41.0, 24.0 and 27.0 months, respectively; *P* = 0.1820), especially between stage II and stage III (Fig. 5a). In the ISS + ARID staging system, 19 patients had stage I, 113 patients had stage II and 15 patients had stage III. A significant reduction of PFS was observed as the stage progressed (median PFS were 41.0, 28.0 and 15.0 months, respectively; *P* = 0.0101) supporting a higher prediction power of ISS + ARID staging system (Fig. 5b). This was also the case for OS. Patients could not be clearly distinguished according to ISS staging (median OS was not reached, 3-year OS was 95.4%, 65.8% and 76.5%, *P* = 0.0659) (Fig. 5c) while there was a significant difference of OS by using ISS + ARID staging (3-year OS was 100%, 75.6% and 61.0%, *P* = 0.0220) (Fig. 5d).

According to the R-ISS, 18 patients were stage I, 89 patients were stage II, and 31 patients were stage III. There was no significant difference in PFS among these three groups (median PFS was 41.0, 24.0 and 33.0 months, respectively; *P* = 0.1891). When using ISS + ARID staging system, 15 patients had stage I, 114 patients had stage II and 9 patients had stage III. ISS + ARID staging displayed better performance in distinguishing PFS as the stage progressed (median PFS was 41.0, 27.0 and 13.0 months, respectively; *P* = 0.0005) (Fig. 5e). Similar findings were obtained for OS. Patients could not be clearly distinguished according to R-ISS staging (median OS was not reached, 3-year OS was 94.1%, 73.6% and 60.6%, *P* = 0.0787) (Fig. 5g), while there was a significant difference of OS by using ISS + ARID staging (3-year OS was 100%, 74.8% and 20.8%, *P* = 0.0015) (Fig. 5h).

### Construction and validation of the nomogram based on ARID mutation

After removing the missing values, we got 136 patients with complete clinical data. Candidate variables were added into Cox proportional hazards regression models of PFS and OS, including age, gender, EMD, subtype, DS staging, ISS staging, R-ISS staging, LDH level, high-risk cytogenetic abnormalities, anemia, renal insufficiency, 1q21 copy numbers, mutation load, ARID family mutations and *TP53* mutations. The final variables were selected based on coefficients and *P*-values of stepwise regression as well as clinical values. Regression coefficients and HRs for the final model variables are presented in Additional file 1: Tables S4 and S5.

**Fig. 6:**
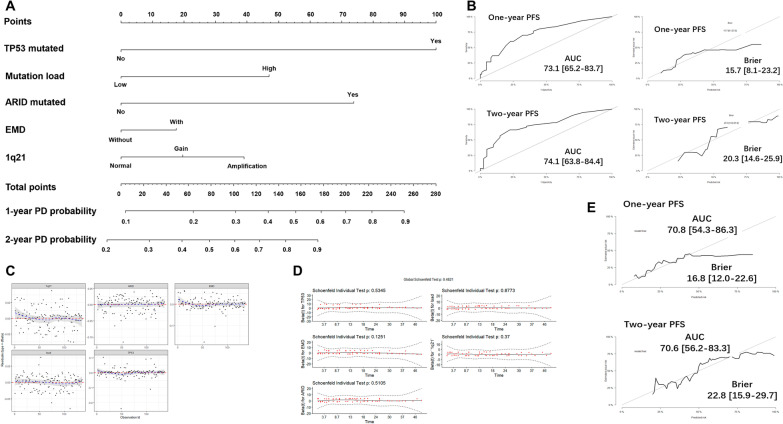
Nomogram model for 1-year and 2-year probability of disease progression. **a** The scores are 100 for harboring TP53 mutations, 47 for having high mutational load, 73 for harboring ARID family mutations, 17.5 for having EMD, 20 for 1q21 gain and 39.5 for 1q21 amplification. The total points are calculated by summing the scores of each variable and the predicted risk corresponding to the total score is the probability of disease progression. **b** The ROC plot shows a good performance of this nomogram for 1-year and 2-year PFS. **c** There is no outlier case detected by Deviance residues test. **d** Schoenfeld residuals test suggests that the Cox models meet the proportional hazards assumption. **e** In the validation sets, the AUC and Brier scores of this model are all satisfactory

We established a nomogram model for PFS including *TP53* mutations, mutation load, ARID family mutations, EMD and 1q21 copy numbers (Fig. 6a). The total points were calculated by summing the scores of each variable, and the predicted risk corresponding to the total score was the probability of PD. The ROC plot showed a good performance of this model in predicting 1-year and 2-year PFS for the AUC were 0.731 and 0.741. The Brier score obtained from the model was 0.157 and 0.203 which confirmed the calibration was acceptable (Fig. 6b). There was no outlier case detected by Deviance residues test (Fig. 6c), and Schoenfeld residuals test suggested that the Cox models met the proportional hazards assumption (Fig. 6d). With internal validation, the AUC (0.708 for 1-year PFS and 0.706 for 2-year PFS) and Brier scores (0.168 for 1-year PFS and 0.228 for 2-year PFS) of this model were all satisfactory (Fig. 6e).

**Fig. 7:**
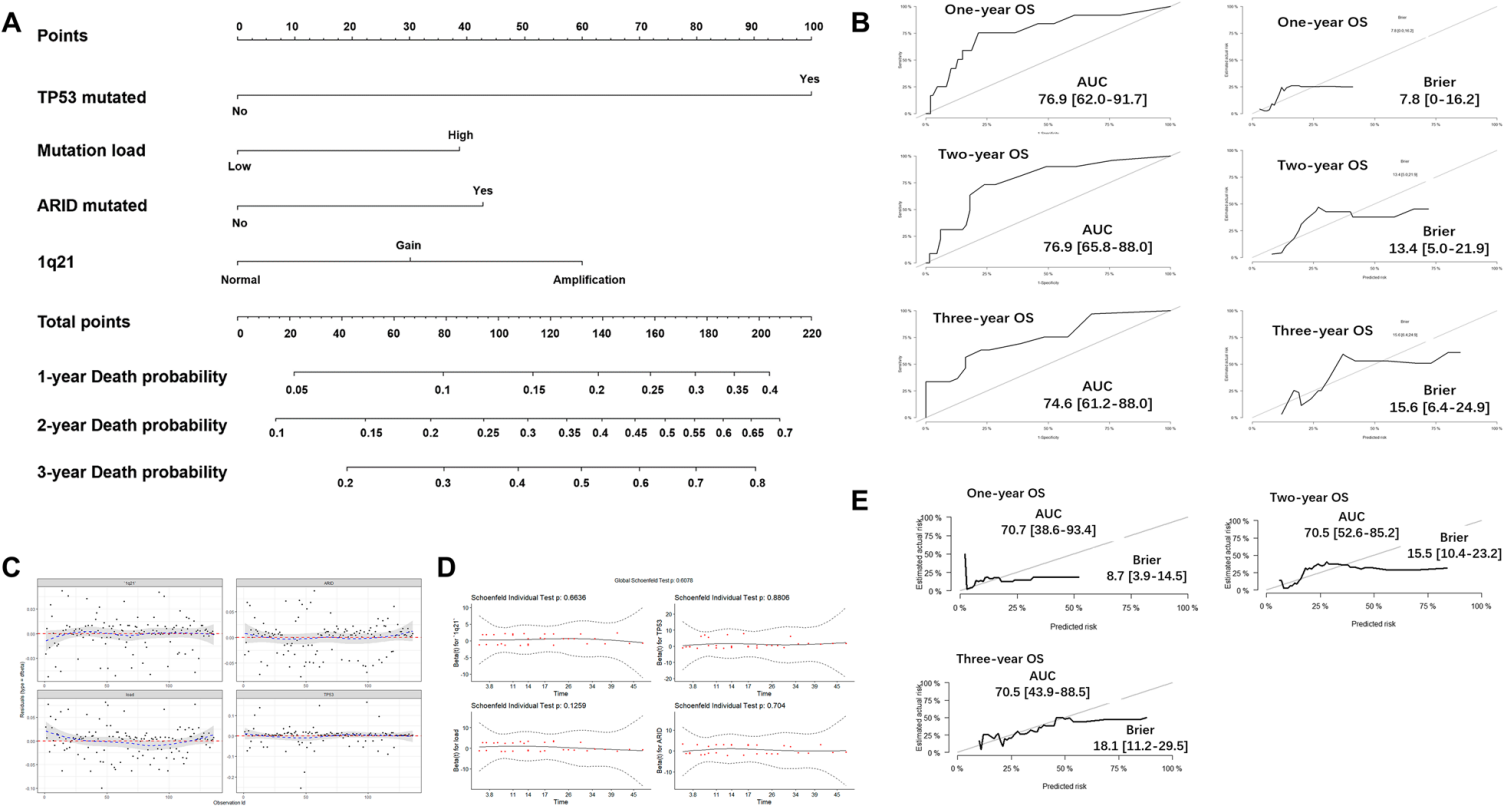
Nomogram model for 1-year, 2-year and 3-year probability of death. **a** The scores are 100 for harboring TP53 mutations, 38 for having high mutational load, 42.4 for harboring ARID family mutations, 30 for 1q21 gain and 60 for 1q21 amplification. The total points are calculated by summing the scores of each variable and the predicted risk corresponding to the total score is the probability of death. **b** The ROC plot shows a good performance of this nomogram for 1-year, 2-year and 3-year OS. **c** There is no outlier case detected by Deviance residues test. **d** Schoenfeld residuals test suggests that the Cox models meet the proportional hazards assumption. **e** In the validation sets, the AUC and Brier scores of this model are all satisfactory

Likewise, we developed a nomogram model for OS by using *TP53* mutations, mutation load, ARID family mutations and 1q21 copy numbers (Fig. 7a). The predicted risk corresponding to the total score was the probability of death. The model performed well in predicting 1-year, 2-year and 3-year OS for the AUC that were 0.769, 0.769 and 0.746. The Brier score obtained from the model was 0.078, 0.134 and 0.156 which confirmed the calibration was acceptable (Fig. 7b). No outliers were identified by Deviance residues test (Fig. 7c) and Schoenfeld residuals test (Fig. 7d). With internal validation, the AUC (0.707 for 1-year OS, 0.705 for 2-year OS and 0.705 for 3-year OS) and Brier scores (0.087 for 1-year OS, 0.155 for 2-year OS and 0.181 for 3-year OS) confirmed this model was eligible for predicting OS (Fig. 7e).

## Discussion

MM is a highly heterogeneous cancer whose development is driven by numerous factors, including cytogenetic abnormalities, changes in the bone marrow microenvironment and aberrant immune regulation [17]. A growing number of studies have revealed that epigenetic alterations, including dysfunction of CRs, play a critical role in the occurrence and progression of MM. Tessoulin et al. [18] found human myeloma cell lines displayed high mutation rates of epigenetic modifiers. The mutation rate of *TET2* (a DNA methylation regulator) was 15%, and the mutation rate of *SETD2* (a chromatin remodeler) was 6%. Ohguchi et al. [19] identified a KDM3A-KLF2-IRF4 axis that maintained myeloma cell survival and dysfunction of KDM3A (a chromatin remodeler) was toxic to MM cells in vitro and in vivo. However, the role of CRs in MM remains poorly studied and relevant clinical studies are particularly lacking. The present study was the first to systematically analyzed the clinical significance of CRs mutations in MM and provided a direction for subsequent mechanistic studies.

*KRAS* was the most commonly mutated gene in our study followed by *NRAS*, which was consistent with existing reports [3–5]. Among the top 15 genes with the highest mutation rate, *KMT2C*, *KMT2D* and *EP300* are CRs. KMT2C/KMT2D are involved in the methylation of H3K4 and p300 (encoded by *EP300*) regulates H3K27 acetylation [9]. It was interesting that KMT2C, KMT2D and p300 could form a complex called K_CD_COMs to activate transcription under normal conditions [16], indicating that the dysfunction of K_CD_COMs might be involved in the pathogenesis of MM. Previous studies demonstrated that mutations affecting components of the K_CD_COMs were associated with the development of other tumors, including breast cancer, lung cancers and B cell lymphomas [20–23], The possible mechanism was considered that dysfunction of K_CD_COMs could promote oncogene expression (e.g., BCL2) and decrease tumor suppressor gene expression (e.g., TP53 and SOCS3) [20, 22, 23]. However, the role of K_CD_COMs in MM is not clear and subsequent functional experiments are needed to be further conducted.

We identified, for the first time, harboring mutations of ARID gene family as a predictor of poor prognosis in MM. It could be incorporated into ISS or R-ISS staging system for the optimal stratification of patients with MM. Subsequently, we constructed nomogram models to predict PFS and OS based on ARID family mutations and the model performed well with good discrimination and calibration. There are seven subfamilies and 15 members in ARID gene family (see Additional file 1: Table S3 for details). All members contain a DNA-binding domain and have the ability to regulate transcription [24]. Much research effort has been focused on the dysfunction of ARID gene family in a variety of tumors. Loss of ARID1A expression was related to poor outcomes in ovarian clear cell carcinoma [25]. Mutations of *ARID5B* might be a potential cofactor in patients with ETV6-linked leukemia predisposition [26]. Frequent deletions of JARID2 promoted the transformation of chronic myeloid malignancies to leukemia [27]. However, the role of this gene family in MM is still unknown, which should be explored in further study.

Currently, there is consensus that chromosomal abnormalities are present before gene mutations during the pathogenesis of myeloma [6]. Therefore, marked correlations were observed between chromosomal abnormalities and gene mutations in MM [5, 28]. We found *FGFR3* mutations were associated with t(4; 14) and TP53 mutations were associated with del(17p), which were in line with other studies [28]. However, the association of gain(1q21) with mutations of *EP300* has not been reported in MM before. Gain/amplification of 1q21 copies is considered a secondary genomic event during MM progression. The incidence of gain(1q21) increased from monoclonal gammopathy of undetermined significance to relapsed MM [29]. So, we speculate that mutations of *EP300* might promote the tumor progression of MM. On the other hand, del(17p) is a well-established marker of poor prognosis in MM, but our results demonstrated the combination of del(17p) and *TP53* mutations could better predict outcomes of MM patients. Patients who had both del(17p) and *TP53* mutations presented with a very poor prognosis and should be paid more attention to by clinicians.

However, there were still some limitations in the present study. It was regretful that we did not perform external validation due to the absence of suitable data. The effectiveness of this model will be externally validated using multi-center data in the future. Moreover, we used the data of normal person in databases but not the same patient’s non-tumor tissue to serve as controls, so we were unable to completely eliminate germline variants. By using strict bioinformatic analysis we tried to minimize the influence of germline variants.

In conclusion, our findings emphasized the importance of CRs mutations in NDMM patients and the mutations affecting K_CD_COMs might promote the development of MM. High mutational load and harboring mutations in the ARID gene family were novel predictors of adverse prognosis in MM. Prognostic models based on 1q21 copies, ARID gene mutations, extramedullary disease, mutational load and TP53 mutations were commendably prognostic evaluation methods for OS and PFS and provided a reference for clinical evaluation.

## Supplementary Information

**Additional file 1**. Supplementary Material.

**Additional file 2**. 387 genes selected for sequencing in this study.

## Data Availability

The data used or analyzed during the current study are available from the corresponding author on reasonable request.

## Abbreviations

AUC: Area under the receiver operator curve
BAM: Binary Alignment Map
BP: Biological process
CC: Cellular component
CR: Chromatin regulator
DS: Durie–Salmon
EMD: Extramedullary disease
GO: Gene ontology
Hb: Hemoglobin
ISS: International Staging System
LDH: Lactate dehydrogenase
MF: Molecular function
MM: Multiple myeloma
NDMM: Newly diagnosed multiple myeloma
NGS: Next-generation sequencing
OS: Overall survival
PD: Disease progression
PFS: Progress-free survival
R-ISS: Revised International Staging System
ROC: Receiver operator curve
SAM: Sequence Alignment/Map
SNP: Single nucleotide polymorphism
