# Supplementary material for: Identification of clinical implications and potential prognostic models of chromatin regulator mutations in multiple myeloma
Source: Clin Epigenetics. 2022 Jul 23;14:93. doi: 10.1186/s13148-022-01314-7 (PMC9308335; doi:10.1186/s13148-022-01314-7)
Supplement: Supplementary file 1 — Additional file 1. Supplementary Material. [file 13148_2022_1314_MOESM1_ESM.docx]

Table S1. Mutational sites of *KMT2C* in our study

| **Sites** | **Number** |
| --- | --- |
| exon1:c.C71T:p.A24V | 1 |
| exon4:c.A527G:p.N176S | 1 |
| exon7:c.G962A:p.S321N | 7 |
| exon7:c.G929C:p.C310S | 1 |
| exon16:c.G2698C:p.G900R | 1 |
| exon18:c.C2944T:p.Q982X | 1 |
| exon33:c.C4984A:p.P1662T | 1 |
| exon36:c.C5792T:p.S1931L | 1 |
| exon36:c.A5888T:p.Y1963F | 1 |
| exon36:c.T6904A:p.S2302T | 1 |
| exon38:c.G7741A:p.V2581I | 1 |
| exon38:c.C9034T:p.Q3012X | 1 |
| exon43:c.A10844G:p.D3615G | 1 |
| exon43:c.G11098A:p.A3700T | 1 |
| exon43:c.A11108T:p.E3703V | 1 |
| exon47:c.A11969G:p.D3990G | 1 |

Table S2. Mutational sites of *KMT2D* in our study

| **Sites** | **Number** |
| --- | --- |
| exon10:c.G1444T:p.A482S | 1 |
| exon28:c.G5908A:p.D1970N | 1 |
| exon28:c.C5921T:p.T1974M | 1 |
| exon31:c.A6682G:p.T2228A | 1 |
| exon31:c.C6740T:p.P2247L | 1 |
| exon31:c.G7036A:p.G2346S | 1 |
| exon34:c.C8764T:p.R2922W | 1 |
| exon34:c.C9326A:p.P3109H | 1 |
| exon39:c.11729_11734del:p.3910_3912del | 3 |
| exon39:c.11750_11758del:p.3917_3920del | 1 |
| exon39:c.G12764A:p.G4255D | 2 |
| exon39:c.C12962T:p.S4321L | 1 |
| exon39:c.C13470A:p.H4490Q | 1 |
| exon42:c.G13924A:p.V4642I | 2 |
| exon42:c.A13981G:p.S4661G | 1 |
| exon45:c.G14339A:p.S4780N | 1 |
| exon48:c.G15512A:p.R5171Q | 1 |

Table S3. Mutational sites of *EP300* in our study

| **Sites** | **Number** |
| --- | --- |
| exon2:c.G189C:p.Q63H | 1 |
| exon2:c.A274G:p.N92D | 1 |
| exon4:c.A1094G:p.N365S | 1 |
| exon4:c.T1150A:p.S384T | 1 |
| exon6:c.G1297A:p.A433T | 1 |
| exon8:c.A1666G:p.M556V | 2 |
| exon10:c.G1936C:p.E646Q | 1 |
| exon10:c.2053+2T>G | 1 |
| exon14:c.A2419G:p.I807V | 1 |
| exon19:c.T3554A:p.I1185K | 1 |
| exon29:c.C4687G:p.L1563V | 1 |
| exon31:c.C5623T:p.P1875S | 2 |
| exon31:c.C5957T:p.P1986L | 1 |
| exon31:c.C6583G:p.Q2195E | 1 |
| exon31:c.A7043G:p.H2348R | 1 |
| exon31:c.A7186G:p.S2396G | 1 |

Table S4. Progression free survival model

| **Variables** | **coef** | ***P* value** | **HR** | **95% CI** |
| --- | --- | --- | --- | --- |
| 1q21 copy numbers | 0.2432 | 0.1216 | 1.275 | 0.937-1.735 |
| Mutation load | 0.5846 | 0.0244 | 1.794 | 1.079-2.985 |
| ARID family mutations | 0.9197 | 0.0003 | 2.508 | 1.521- 4.138 |
| *TP53* mutations | 1.2443 | 0.0008 | 3.471 | 1.679-7.175 |
| EMD | 0.2182 | 0.4577 | 1.244 | 0.699-2.212 |

HR, hazard ratio; CI, confidence interval; EMD, extramedullary disease

Table S5. Overall survival model

| **Variables** | **coef** | ***P* value** | **HR** | **95% CI** |
| --- | --- | --- | --- | --- |
| *TP53* mutations | 1.2613 | 0.0081 | 3.5299 | 1.387-8.985 |
| Mutation load | 0.4884 | 0.1897 | 1.6297 | 0.785-3.381 |
| 1q21 copy numbers | 0.3789 | 0.1007 | 1.4606 | 0.929-2.296 |
| ARID family mutations | 0.5395 | 0.14282 | 1.7152 | 0.834-3.530 |

HR, hazard ratio; CI, confidence interval; EMD, extramedullary disease

Table S6. Members of the ARID family

| **Subfamily** | **Member** |
| --- | --- |
| ARID1 | ARID1A, ARID1B |
| ARID2 | ARID2 |
| ARID3 | ARID3A, ARID3B, ARID3C |
| ARID4 | ARID4A, ARID4B |
| ARID5 | ARID5A, ARID5B |
| JARID1 | JARID1A/KDM5A, JARID1B/KDM5B, JARID1C/KDM5C, JARID1D/KDM5D |
| JARID2 | JARID2 |
