# Supplementary material for: Identification of clinical implications and potential prognostic models of chromatin regulator mutations in multiple myeloma
Source: Clin Epigenetics. 2022 Jul 23;14:93. doi: 10.1186/s13148-022-01314-7 (PMC9308335; doi:10.1186/s13148-022-01314-7)
Supplement: Supplementary file 2 — Additional file 2. 387 genes selected for sequencing in this study. [file 13148_2022_1314_MOESM2_ESM.pdf]

ADAM29
ADAR
ADAMTS9
AHNAK2
AKT1
ALK
ANK2
AP3B2
APC
APOBEC1
APOBEC2
APOBEC3A
APOBEC3B
APOBEC3C
APOBEC3D
APOBEC3F
APOBEC3G
APOBEC3H
APOBEC4
ARID1A
ARID1B
ARID2
ARID3A
ARID3B
ARID3C
ARID4A
ARID4B
ARID5A
ARID5B
ASAP2
ASB15
ASCC3
ASXL1
ATF7IP
ATM
ATN1
ATP13A4
ATP2A1
ATP2A2
ATP2A3
ATR
ATXN1
ATXN3
B2M
BAGE2
BCL2
BCL6
BCOR
BIRC2
BIRC3
BLM

BRAF
BRCA1
BRCA2
BRIP1
BTK
BUB1B
C11orf30
C2orf50
CACNA1I
CARD11
CARD9
CASP8
CBL
CCDC144NL
CCDC148
CCDC39
CCNB1
CCND1
CCND2
CCND3
CCNT1
CD27
CD40
CD79A
CD79B
CDC73
CDK4
CDK6
CDK7
CDKN1B
CDKN2A
CDKN2C
CELA1
CELSR1
CHD8
CHEK2
CIC
CIDEC
CMYA5
COL2A1
CPXCR1
CRBN
CREBBP
CSMD1
CSMD3
CSNK2A1
CTNNAL1
CUL4A
CUL4B
CXCR4
CYLD

CYP26A1
DDB1
DDX11
DDX3X
DEFB112
DENND4A
DICER1
DIS3
DMD
DNAH11
DNAH5
DNAH9
DNMT3A
DTX1
DUSP2
E2F4
EGFR
EGR1
EIF4G2
EP300
EP400
EPHA3
ERBB3
ERN1
ETV1
ETV6
EZH2
FAM166B
FAM46C
FANCA
FANCD2
FAT1
FAT2
FAT3
FAT4
FBN2
FBXW7
FGFR1
FGFR2
FGFR3
FLT1
FLT3
FN1
FOXA2
FOXP1
FRG1B
FRYL
FTSJ3
GATA6
GNAS
GPC5

GPR180
GRB2
HDAC2
HECW1
HIST1H1C
HIST1H1D
HIST1H1E
HIST1H2AG
HLA-DQA1
HMCN1
HOXA9
HRAS
IARS2
ID3
IDH1
IDH2
IDH3A
IFNGR2
IGF1R
IGF2R
IGLL5
IKZF1
IKZF3
IL6
IL6R
IL6ST
IRF4
JAK2
JARID2
KDM5A
KDM5B
KDM5C
KDM5D
KDM6A
KDM6B
KDR
KIAA1462
KLHL6
KMT2A
KMT2B
KMT2C
KMT2D
KMT2E
KRAS
LAMA3
LEPR
LINGO1
LOC96610
LRP1B
LRP2
LRRK2

LTB
LTBR
LTN1
LYST
MAF
MAFB
MAGED1
MAGI1
MAML2
MAMLD1
MAP2K2
MAP2K4
MAP3K1
MAP3K14
MAP3K4
MAP3K9
MAP4K4
MAPK9
MAST4
MAX
MGA
MYC
MDM4
MECOM
MED12
MEGF6
MIA2
MKI67
MLH1
MPDZ
MPEG1
MPL
MPRIP
MRE11A
MUC16
MUC17
MUC2
MUC4
MUTYH
MYCN
MYD88
NBPF1
NCKAP5
NCOR1
NCOR2
NEB
NF1
NFE2L2
NFKB1
NFKB2
NFKBIA

NFKBIB
NOTCH1
NOTCH2
NOTCH3
NOTCH4
NR3C1
NRAS
NTRK2
NXF1
OBSCN
ODZ2
OR4C3
PBRM1
PCLO
PDGFRB
PDIA4
PEG3
PI4KA
PIK3C2G
PIK3CA
PIK3CB
PKHD1
PLCB4
PLCE1
PNRC1
POT1
PPM1D
PRDM1
PRDM16
PRDM9
PRKD2
PRUNE2
PSMA1
PSMB5
PSMB8
PSMB9
PSMD1
PSMG2
PTEN
PTPN11
PTPRD
PTPRK
PTPRT
PTPRZ1
RAD18
RAG2
RAPGEF4
RASA2
RB1
RET
RIPK1

RIPK4
RNF213
ROBO1
ROBO2
ROS1
RPL11
RPL14
RPL17
RPL5
RUNX1
RUNX2
RYR2
RYR3
SALL2
SCN9A
SETD2
SF3B1
SHANK2
SHC1
SI
SLCO4A1
SMAD3
SMARCB1
SMPDL3A
SMURF1
SNX7
SOX21
SOX9
SP140
SPEN
SPOP
SPTAN1
SPTB
SQLE
SRF
SRRM5
SRSF1
SRSF2
STAT3
SURF1
SYNE1
TBP
TCF3
TENM1
TET2
TGDS
TGFBR2
TIAM1
TLR4
TNFRSF13B
TNFRSF21

TNFSF9
TNIK
TOP1
TP53
TRAF2
TRAF3
TRAF3IP1
TRIP12
TSHZ1
TSHZ3
TTN
U2AF1
U2AF2
UNC80
USH2A
USP29
USP7
USP9X
VCAN
VSIG8
WHSC1
WWOX
XBP1
XIRP2
XPO1
YAP1
ZFHX3
ZFHX4
ZNF598
ZRSR2
